# *Pestivirus* infection in sheep and goats in Northern Saudi Arabia

**DOI:** 10.5455/javar.2025.1001

**Published:** 2025-12-30

**Authors:** Yahia Hassan Ali, Intisar Kamil Saeed, Muaz Magzob Abdellatif, Anwar A Alsharari, Medhat Ahmed Abu-Tahon1, Ahmad M Abdel-Mageed, Ali M. S. Eleragi

**Affiliations:** 1 Department of Biological Sciences, College of Science, Northern Border University, Arar, Saudi Arabia; 2 National Center for the Prevention and Control of Plant Pests and Animal Diseases, Sakaka, Saudi Arabia; 3 Department of Pathology and Diagnosis, Central Veterinary Research Laboratory, Soba, Khartoum, Sudan; 4 Department of Microorganisms and Clinical Parasitology, College of Medicine, University of Bisha, Bisha, Saudi Arabia

**Keywords:** *Pestivirus*, Sheep, Goats, Risk factors, Saudi Arabia

## Abstract

**Objective:**

The current research aims to elucidate the recent situation regarding *Pestivirus* infection among different age groups, sexes, and breeds of goats and sheep in northern Saudi Arabia.

**Materials and Methods:**

850 serum samples representing 594 sheep and 256 goats were collected at the slaughterhouse in Rafha city.

**Results:**

Using enzyme-linked Immune Assay, the overall detected seroprevalence in sheep and goats was 24.5%; it was 30.5% in goats and 21.9% in sheep. The detected seroprevalence was higher in goats than in sheep, in females than in males, and in adults than in young. According to the breeds, the highest prevalence (47%) was observed in the Naime breed. Pearson chi-square analysis revealed a significant correlation between infection and animal species (*p* < 0.008), sex (*p* < 0.003), and breed (*p* < 0.000).

**Conclusion:**

The prevalence of *Pestivirus* in sheep and goats appears to be increasing in the Northern region and throughout the country.

## Introduction

Flaviviridae is an Ribonucleic Acid (RNA), single-stranded, positive-sense virus family. It includes the *Pestivirus* genus, which includes bovine viral diarrhea virus (BVDV). The *Pestivirus* genus includes four species: bovine viral diarrhea virus type 1 and 2 (BVDV-1, BVDV-2) of cattle, border disease virus (BDV) of small ruminants, and classical swine fever virus of pigs [1]. *Pestivirus* taxonomy update of 2017, nominated 11 species of *Pestivirus* from A to K [2]. Currently, BVDV-1, BVDV-2, and HoBiPev are classified into the following species:* Pestivirus bovis*, *Pestivirus tauri*, and *Pestivirus brazilense* [3]. Mainly, BVDV infection affects the reproductive system, leading to immune and reproductive dysfunction and clinical signs including slow fetal growth, decreased productivity, diarrhea, and respiratory symptoms [4,5]. A review of BVD, encompassing 59 manuscripts, concluded that the disease has a high prevalence, which was reported in the Middle East, whereas the lowest prevalence was observed in Asia[1] . In Europe, the infection with BDV is widely distributed, as seen in Ireland[6] , as well as in goats in Italy[7,8] and Poland[9] . In Africa, seropositivity for BDV has been reported in sheep in Algeria[10] and Morocco[11] , in goats in Egypt[12] , and in both sheep and goats in Sudan[13] . It was reported in China in goats[14] and sheep[15] , as well as in sheep and goats in Indonesia[16] and Iraq[17,18] , and in goats in Korea[19] . In Saudi Arabia, *Pestivirus* has been detected in Al-Ahsa, located in the Eastern region, in sheep and goats[20] and in camels and goats[21] . Additionally, it has been detected in sheep and goats in Rafha and Hail, both in the Northern region[22] . The present research aims to elucidate the recent situation of *Pestivirus* within sheep and goats of different sexes, ages, and breeds in the North of Saudi Arabia through detecting antibodies against the virus.

## MATERIALS AND METHODS

### Ethical approval

Animals were sampled during slaughter in slaughterhouses in accordance with ethical values, and the procedure was authorized by the Local Committee of Bioethics (HAP-09-A-043) at Northern Border University, KSA.

### Area of study

**Figure 1.**
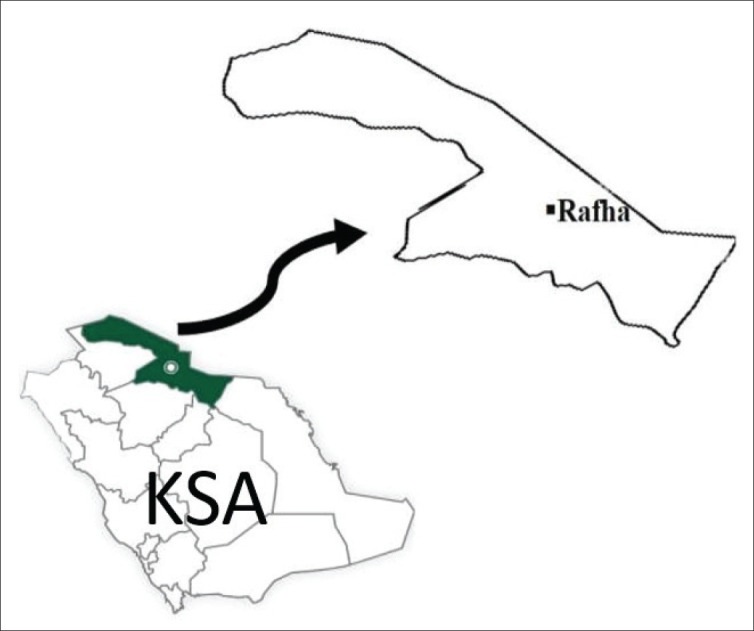
Map showing Rafha city in the northern border region of Saudi Arabia, where samples were collected.

The study was conducted in Rafha town, north of Saudi Arabia (Fig. 1). This serological survey aimed to elucidate the current prevalence of *Pestivirus* in sheep and goats, as well as its associated risk factors in Northern Saudi Arabia. It is based on the previous studies in the country [20–22].

### Sample size calculation

The formula described by Thrusfield et al. [23] was used to estimate the sample size, with an intended absolute precision of 5% and a 95% confidence interval. The expected prevalence (11.1%) was estimated based on a previous study [20]. Where *p* is the prevalence expected, while d is the target precision, and *n* is the needed samplesize.

Upon replacing each value, *n* = 170 is obtained. The sample size was multiplied by five (*n* = 850) to enhance precision.

### Sample collection

As small ruminant farms in the city are very limited, samples were randomly collected from selected sheep and goats that were kept for slaughter. Eight hundred and Fifty Sera (sheep, *n* = 594; goats, *n* = 256) were taken randomly during animal slaughtering at the slaughterhouse in Rafha. Data on the age, sex, and breeds of the sample animals were collected and analyzed (Table 1).

Samples were collected post-mortem during routine slaughtering processes at licensed abattoirs. No live animals were handled or subjected to experimental procedures. The collection of samples did not interfere with the standard operations of the abattoirs, and all procedures adhered to local and international regulations regarding animal welfare and research ethics.

### Detection of Pestivirus antibodies

The collected sera were screened for detection of *Pestivirus* antibodies using competitive ELISA kits (Blocking ELISA, INgezim *Pestivirus* Compac), with the *Pestivirus* non-structural proteins NS2-3 (p80/p125) specific monoclonal antibodies peroxidase conjugate obtained through Immonologia Y Genetica Aplicada, S.A. C./ Hnos. Company at Garcia, Noblejas, 39 28037—Madrid, Spain, and used according to the provided protocol (Prod Ref: 12. DVD.K3). The positive control ODs are < 0.4, and the negative control ODs are < 0.8. Cutoff was calculated as follows: positive Cut Off = Negative control OD × 0.5, while Negative Cut Off = Negative control OD × 0.55.

### Statistical analysis

Data has been recorded, categorized, and stored in a spreadsheet in Microsoft Excel. Then transferred to version 27 of IBM's SPSS^®^. Saved data, including *Pestivirus* infection, species, sex, age, and breed, were summarized using descriptive and analytical statistics.

### The pearson chi-square

The pearson chi-square test was used for estimating the correlation between *Pestivirus* infection, with the noted variables. At *p* < 0.05, with a 95% confidence level for the analyses, the statistical significance was determined.

### The Logistic regression analysis

To determine the best-fitting model for understanding the relationship between *Pestivirus* infection and the recorded variables, binary logistic regression was used.

### The model coefficients

To assess the model’s fitness, the omnibus test is employed; we utilized the ratio likelihood, which follows a chi-square distribution. To test the null hypothesis, the Hosmer–Lemeshow test was used. A chi-square statistic is done by comparing the frequencies observed to the generalized linear model-predicted ones. For the analysis, the statistical significance was set at *p* < 0.05, with a 95% confidence level.

## Results

### Pestivirus seroprevalence

**Figure 2.**
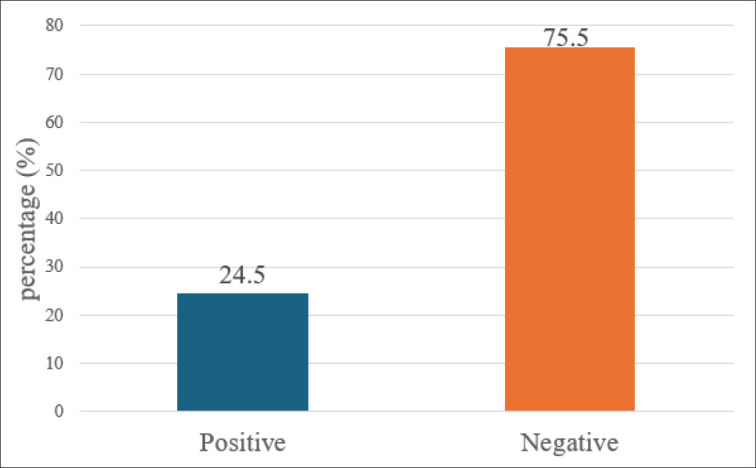
Prevalence of *Pestivirus* among small ruminants as detected by ELISA.

**Figure 3.**
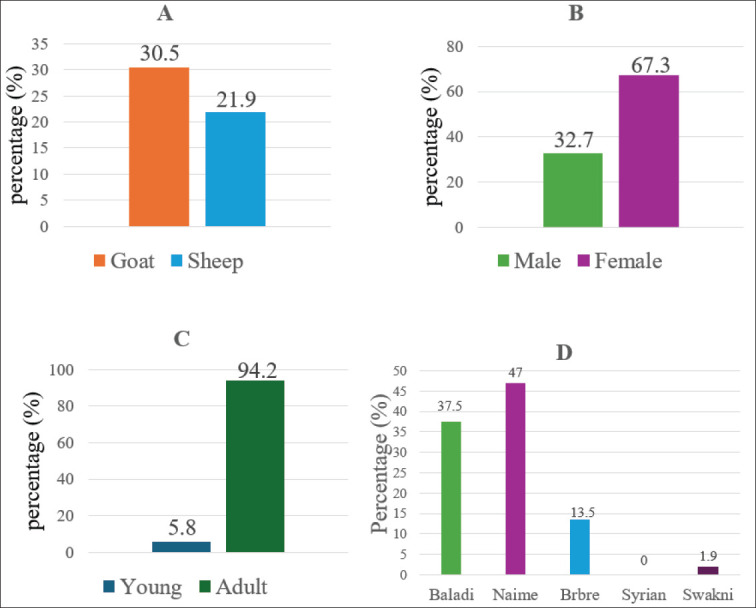
Prevalence of *Pestivirus* as detected by ELISA, according to species (A), sex (B), age (C), and breed (D) investigated.

Tested sera showed 24.5% overall *Pestivirus* seroprevalence. It was 30.5% in goats and 21.9 in sheep (Figs. 2, 3).

### Pestivirus seroprevalence distribution within sex, young, and adult animals

*Pestivirus* variable seroprevalence was noticed; among goats, the higher prevalence was found in females (76.9%) and adults (100.0%), whereas in sheep it was recorded among females (61.5%) and adults (90.8%) (Tables 2, 3).

**Table 1.** Descriptive analysis of the study animals according to species, sex, age and breed.

Species		Male	Female	Total	Young	Adult	Total
Goat	Count	101	155	256	6	250	256
	%	39.5	60.5	100.0	2.3	97.7	100.0
Sheep	Count	252	342	594	50	544	594
	%	42.4	57.6	100.0	8.4	91.6	100.0
Total	Count	353	497	850	56	794	850
	%	41.5	58.5	100.0	6.6	93.4	100.0
Species		Baladi	Naime	Brbre	Syrian	Swakni	Total
Goat	Count	214	0	0	42	0	256
	%	83.6	0.0	0.0	16.4	0.0	100.0
Sheep	Count	0	476	98	0	20	594
	%	0.0	80.1	16.5	0.0	3.4	100.0
Total	Count	214	476	98	42	20	850
	%	25.2	56.0	11.5	4.9	2.4	100.0

**Table 2.** Prevalence of BVD according to species, sex and age as tested by ELISA.

Species		Male	Female	Total	Young	Adult	Total
Goat	Count	18	60	78	0	78	78
	%	23.1	76.9	100.0	0.0	100.0	100.0
Sheep	Count	50	80	130	12	118	130
	%	38.5	61.5	100.0	9.2	90.8	100.0
Total	Count	68	140	208	12	196	208
	%	32.7	67.3	100.0	5.8	94.2	100.0

**Table 3.** Prevalence of BVD according to breed as tested by ELISA.

Species		Baladi	Naime	Brbre	Syrian	Swakni	Total
Goat	Count	78	0	0	0	0	78
	%	100.0%	0.0%	0.0%	0	0.0%	100.0%
Sheep	Count	0	98	28	0	4	130
	%	0.0%	75.4%	21.5%	0	3.1%	100.0%
Total	Count	78	98	28	0	4	208
	%	37.5%	47.1%	13.5%	0	1.9%	100.0%

**Table 4.** Pearson correlation between the BVD, species, sex, age and breed.

Variable	Parameter	BVD	Spp.	Sex	Age	Breed
BVD	Pearson Correlation	1	−0.092**	0.102**	0.019	−0.139**
	Sig. (2-tailed)		0.008	0.003	0.584	0.000
Spp.	Pearson Correlation	−0.092**	1	−0.028	−0.112**	0.403**
	Sig. (2-tailed)	0.008		0.421	0.001	0.000
Sex	Pearson Correlation	0.102**	−0.028	1	0.084*	−0.297**
	Sig. (2-tailed)	0.003	0.421		0.014	0.000
Age	Pearson Correlation	0.019	−0.112**	0.084*	1	−0.065
	Sig. (2-tailed)	0.584	0.001	0.014		0.056
Breed	Pearson Correlation	−0.139**	0.403**	−0.297**	−0.065	1
	Sig. (2-tailed)	0.000	0.000	0.000	0.056

**. Correlation is significant at the 0.01 level (2-tailed); *. Correlation is significant at the 0.05 level (2-tailed).

**Table 5.** *Pestivirus*infection pearson chi-square analysis within species, sex, age, breed.

Parameter	Species	Sex	Age	Breed
Pearson chi-square	7.131^a^	8.857^a^	1.237	35.209
*df*	1	1	2	4
Asymptotic significance	0.008	0.003	0.539	0.000

*df*, the degree of freedom.

^a^In the chi-square test each table has expected a less than 5 count without affecting the accuracy of the test.

**Table 6.** The statistical significance of included variables in the fitted model for the *Pestivirus*infection prevalence.

Variable	B	S.E.	Wald	*df*	Sig.	Exp(B)	95% C.I. for EXP(B)	
							Lower	Upper
sex	0.333	0.174	3.683	1	0.055	1.395	0.993	1.960
breed	−0.376	0.110	11.760	1	0.001	0.687	0.554	0.851
Constant	−0.976	0.182	28.858	1	0.000	0.377

S.E.: The standard error; *df*: The degree of freedom; Sig.: significance; CI: confidence interval.

### Variation of Pestivirus seroprevalence within different breeds

*Pestivirus* seroprevalence detected showed some variations in different breeds; the highest observed figures in goats were found in the Baladi breed (100%), and in sheep, in the Naime breed (75.4%) (Tables 2,3 & Fig. 3).

### Age distribution

**Figure 4.**
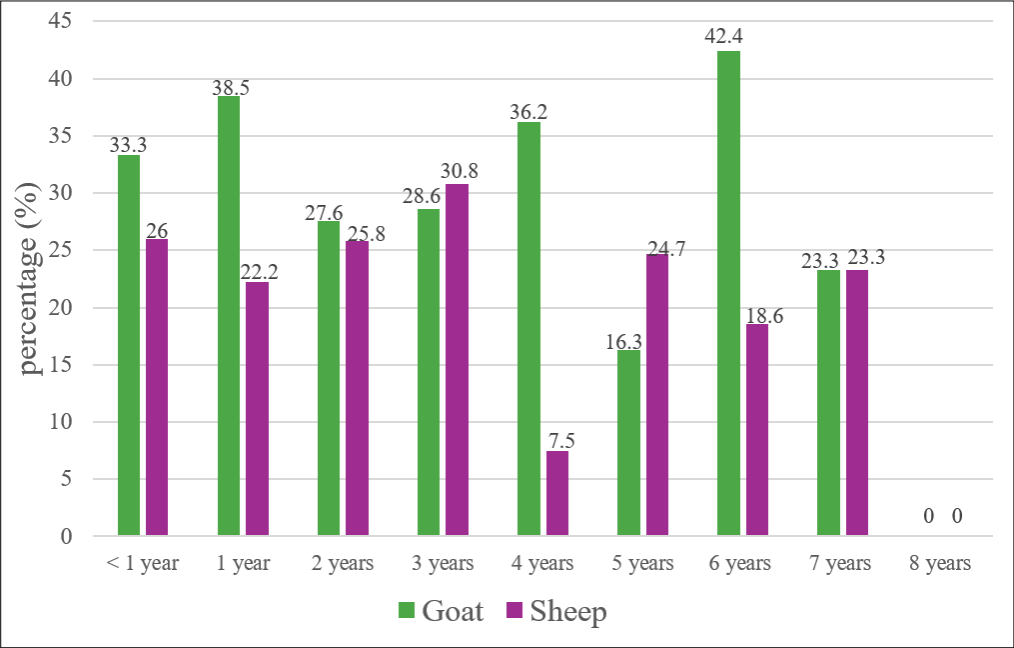
Prevalence of *Pestivirus* as detected by ELISA, according to the age of the animals investigated.

Age-wise, the higher prevalence rate was recorded in 6-year-old goats (42.4%) and 1-year-old sheep (38.5%) (Fig. 4).

### The pearson chi-square

A significant association was indicated using pearson chi-square analysis for *Pestivirus* infection with species (*p* < 0.008), sex (*p* < 0.003), and breed (*p* < 0.000) (Table 4,5).

### The multivariate analysis

The significance of the fully successful model (*p* = 0.000) was confirmed using the Omnibus test. The fitness of the data to the model (*p* = 0.348) was indicated using the Hosmer and Lemeshow test.

### Logistic regression model 

Analysis revealed that the logistic regression model can be fit as:

ln (*odds*) = −0.976 + 0.333 × sex −0.376 × breed

Wald statistics revealed that significant predictors included sex and breed, while non-significant predictors were removed, including species and age. In the full model, breed remained a strong predictor (*p* = 0.001), while sex showed a marginal effect (*p* = 0.055). The odds of the outcome were 1.4 times higher for one sex and 31% lower for a particular breed (Table 6).

## Discussion

*Pestivirus* infections are known as significant disease agents in small and large ruminants. In a review, 34,452 sheep and goat sera from 31 datasets revealed an 8.6% prevalence of BVDV infection; the highest prevalence (26%) was found in South America [24]. In this research, 24.5% overall *Pestivirus* seroprevalence was detected in goats and sheep. Our results were higher than those previously reported (18%) in Rafha and Hail in the northern region of Saudi Arabia [22] and (17%) in Al-Ahsa in the eastern region of Saudi Arabia [20]. This could be attributed to the further spread of the infection due to weak control measures, as no vaccination programs or hygienic measures are being applied. Variable *Pestivirus* seroprevalence in sheep and goats has been reported: 31% in Sudan [13]; in Europe, 1.7% of animals and 17% of flocks were seropositive in Ireland [6], 8.6% in reviewed reports from 24 countries [24], and 3.5% in Australia [25]. The results indicated a wider spread of *Pestivirus* infection generally in Saudi Arabia and specifically in the Northern region of the country. This could be attributed to the weak management system due to the negligence of the role of *Pestivirus* in animal production.

This study showed 22% seroprevalence in sheep, which is lower than the previously reported one (29%) at the same province (Rafha) but higher than that observed (13%) in another province (Hail) in the Northern region of Saudi Arabia [22]. A very close seroprevalence (24%) was detected in Eastern Saudi Arabia [20] and 25% in Turkey [26]. A higher seropositivity rate (73%) for BDV was reported in Algeria [10]. Results for border disease (23%), similar to this study, were detected [27]. However, in Turkey, a 51% seropositivity rate for BVDV was reported [28]. During an outbreak of *Pestivirus* in sheep in Western Sudan, 48% seroprevalence was reported [29], while 39% of screened sheep from different localities tested positive [13]. Lower *Pestivirus* prevalence of 5.6% was found in the USA [30], 8.5% in Greece [31], and 1.7% in Ireland [6]. This is most probably due to the strict hygienic measures adopted there.

In goats, the prevalence of *Pestivirus*, as reported in 31 datasets reviewed from 24 countries, was 8.7% [24]. The *Pestivirus* seroprevalence detected in goats in this study was 30.5%, which is higher than the results of previous reports in Saudi Arabia: 17% in Hail and 16% in Rafha in the northern region [22]. In the Eastern region, the rates were 13% [20] and 4% [32]. In Italy, a very high seroprevalence in goats (91%) was reported [7], while 33% of 57 goat farms were seropositive [8]. Our results indicated that *Pestivirus* infection in goats was widespread in northern Saudi Arabia. As seen in sheep, this is mostly due to the weak hygienic measures adopted.

Within species, reported *Pestivirus* seroprevalence in many studies was found to be higher in sheep than goats in a review of 31 datasets collected from 24 countries [24] and in Rafha and Hail in the Northern region of Saudi Arabia [22]. The same observation was reported in Eastern Saudi Arabia [20], Sudan [13], Iraq [18], and Greece [31]. Unlike these reports, we found that the seroprevalence in goats was higher than in sheep.

Pearson correlation showed in Table 4 that *Pestivirus* infection significantly correlated with animal species, which agreed with previous reports in Eastern Saudi Arabia [21] and Northern Ireland [6]. This may be due to the lower number of sampled goats, which were likely collected from the same flock, as seen in Ireland [6].

Concerning sex, *Pestivirus* seroprevalence detected in this study in both sheep and goats was found to be higher in females than in males. Statistical analysis revealed that seroprevalence was significantly correlated with the sex of the species; a similar trend was observed in Eastern Saudi Arabia [20]. This could be attributed to the close association between *Pestivirus* infection and reproductive problems, which are more likely to be encountered in females than in males, given that most males are slaughtered for meat production at a young age. Regarding breed, variable *Pestivirus* seroprevalence was also documented between breeds in this study; the highest prevalence was observed in the Naime breed, whereas the lowest was seen in the Barbari breed.

In this study, the higher rate of infection was reported in 3-year-old sheep and 6-year-old goats. Variable results in different age groups were documented. In Turkey, the highest BVDV seropositivity was detected in 5-year-old sheep (66%), followed by 4-year-olds, 3-year-olds, 2-year-olds, and then 1-year-olds [28].

The variability of *Pestivirus* seroprevalence within sheep and goats among different age groups was documented; our results showed higher seroprevalence in adult animals than in young ones. However, correlation analysis exhibited no association between the infection and age; the same picture was observed in Eastern Saudi Arabia in sheep and goats [20] and in cattle [32]. Increased seroprevalence with older age groups was reported in Turkey [26,28]and in different countries [24], including India [33]. A statistically significant relationship was found between the age groups. Age was identified as an important risk factor associated with *Pestivirus* seropositivity [26]. BVDV prevalence is significantly different among age groups, with the highest prevalence (32%) in those over 2 years old [24]. In eastern Saudi Arabia, a significant correlation was observed between age and BDV seroprevalence [20]. In a more recent study, a weak association was found between BVDV seroprevalence, age, and the breed of camels [21]. A significant difference was observed for BVDV seropositivity with age in cattle in eastern Saudi Arabia, with higher seropositivity in the 2- to 4-year age group and higher BVDV positivity in the 4-year age group compared to the 2- to 4-year age group [32]. BVDV seroprevalence was higher in the 1-2 year and > 2-year age groups than in the 6-month-1-year age group in sheep and goats in India [33]. In the present study, Higher seropositivity was observed in 6-year-olds, followed by 4-year-olds and then 1-year-olds in goats, whereas in sheep, higher results were detected in 3-year-olds, followed by < 1 year, 2-year-olds, and then 5-year-olds. Although some increase in seropositivity was observed with older age, correlation analysis exhibited no association between infection and age. This is expected since samples were randomly collected at slaughterhouses, not herds or farms.

Analysis exhibited a significant association between the infection and breed, as reported in Eastern Saudi Arabia. However, the highest prevalence was seen in the Awassi breed there [20]. It was also the second most affected breed in Turkey [28].

## Conclusion

It was concluded that the infection of *Pestivirus* in sheep and goats in Northern Saudi Arabia is increasing. A significant association was found between the spread of the infection and the species, sex, and breed of affected animals. Detailed research on the molecular level of *pestivirus* circulating in goats and sheep is highly recommended.

## List of abbreviations

BVDV, bovine viral diarrhea virus; ELISA, Enzyme-linked Immune Assay; nNS, non-structural proteins; ODs, optical density; RNA, Ribonucleic Acid.
